# Nutrients and Foods Recommended for Blood Pressure Control on Twitter in Japan: Content Analysis

**DOI:** 10.2196/49077

**Published:** 2024-06-20

**Authors:** Marina Terada, Tsuyoshi Okuhara, Rie Yokota, Takahiro Kiuchi, Kentaro Murakami

**Affiliations:** 1 Department of Health Communication Graduate School of Medicine The University of Tokyo Tokyo Japan; 2 Department of Health Communication School of Public Health Graduate School of Medicine The University of Tokyo Japan; 3 Department of Medical Communication School of Pharmacy and Pharmaceutical Sciences Hoshi University Tokyo Japan; 4 Department of Social and Preventive Epidemiology School of Public Health The University of Tokyo Tokyo Japan

**Keywords:** Twitter, food, nutrition, misinformation, salt, content analysis, hypertension, blood pressure, sodium, salt reduction

## Abstract

**Background:**

Management and prevention of hypertension are important public health issues. Healthy dietary habits are one of the modifiable factors. As Twitter (subsequently rebranded X) is a digital platform that can influence public eating behavior, there is a knowledge gap regarding the information about foods and nutrients recommended for blood pressure control and who disseminates them on Twitter.

**Objective:**

This study aimed to investigate the nature of the information people are exposed to on Twitter regarding nutrients and foods for blood pressure control.

**Methods:**

A total of 147,898 Japanese tweets were extracted from January 1, 2022, to December 31, 2022. The final sample of 2347 tweets with at least 1 retweet was manually coded into categories of food groups, nutrients, user characteristics, and themes. The number and percentage of tweets, retweets, and themes in each category were calculated.

**Results:**

Of the 2347 tweets, 80% (n=1877) of tweets mentioned foods, which were categorized into 17 different food groups. Seasonings and spices, including salt, were most frequently mentioned (1356/1877, 72.2%). This was followed by vegetable and fruit groups. The 15 kinds of nutrients were mentioned in 1566 tweets, with sodium being the largest proportion at 83.1% (n=1301), followed by potassium at 8.4% (n=132). There was misinformation regarding salt intake for hypertension, accounting for 40.8% (n=531) of tweets referring to salt, including recommendations for salt intake to lower blood pressure. In total, 75% (n=21) of tweets from “doctors” mentioned salt reduction is effective for hypertension control, while 31.1% (n=74) of tweets from “health, losing weight, and beauty-related users,” 25.9% (n=429) of tweets from “general public,” and 23.5% (n=4) tweets from “dietitian or registered dietitian” denied salt reduction for hypertension. The antisalt reduction tweets accounted for 31.5% (n=106) of the most disseminated tweets related to nutrients and foods for blood pressure control.

**Conclusions:**

The large number of tweets in this study indicates a high interest in nutrients and foods for blood pressure control. Misinformation asserting antisalt reduction was posted primarily by the general public and self-proclaimed health experts. The number of tweets from nutritionists, registered dietitians, and doctors who were expected to correct misinformation and promote salt reduction was relatively low, and their messages were not always positive toward salt reduction. There is a need for communication strategies to combat misinformation, promote correct information on salt reduction, and train health care professionals to effectively communicate evidence-based information on this topic.

## Introduction

Hypertension is a modifiable risk factor for cardiovascular disease and premature death [1,2]. In 2019, the global age-standardized prevalence of hypertension in adults aged 30-79 years was reported to be 32% in women and 34% in men [3]. In Japan, the prevalence of hypertension in adults aged 20 years or older was 24.9% in women and 29.9% in men in 2019 [4]. As more than 20% of adults are estimated to have hypertension, its prevention and management remain important public health issues [3].

One of the modifiable preventive factors for hypertension is healthy dietary habits such as reduced salt intake and increased potassium intake [1,5], in addition to other factors such as engaging in moderate physical activity and maintaining normal body weight [5]. The National High Blood Pressure Education Program [5] and the Japanese Society of Hypertension [6] have established dietary and nutrient intake guidelines to prevent and manage hypertension. However, the amount of salt and vegetable intake recommended for blood pressure reduction in Japanese adults has not been achieved in adults aged 20 years or older in 2019 [4].

Our previous study found that “nutrient and hypertension” is one of the most frequently searched keywords in Google among information related to nutrients from 2017 to 2022 [7], indicating high public interest in digital information on diet and hypertension. Furthermore, in a Japanese national survey, between 15.4% and 39.3% of women aged 20-49 years and 10.8%-17.6% of men in the same age group reported that social media was a source of information that influenced their eating behavior in 2019 [4]. Social media is a platform for information on which people post food and diets [8]. Specifically, Japan ranks as the second largest active Twitter (subsequently rebranded X) user, with 69 million users as of 2024 [9]. Twitter is an important communication channel, as more than 40% of the Japanese population uses Twitter [10], and 38.2% of individuals in Japan with hypertension or high blood pressure engage with social media in 2020 [11]. Therefore, we speculate that Twitter users are exposed to information that may influence their eating behaviors.

In a study assessing 550,338 English tweets related to cardiovascular diseases from 2009 to 2015, there were 23,459 tweets regarding hypertension [12], and 43% of 500 manually coded tweets were about treatment and control of hypertension, including diet [12]. A random sample of 1251 tweets from 2009 to 2015 regarding cardiovascular disease also reported that 8% of the tweets mentioned prevention including dietary advice for blood pressure and diabetes control [13]. Studies analyzing information related to hypertension on social media platforms such as Twitter and Facebook reported that common themes included “awareness,” “risk factors,” and “treatment and management” [12-14]. However, it is unclear what types of nutrients and foods are recommended, particularly on Twitter, for the prevention and control of hypertension. A content analysis of 298 tweets related to heart failure and nutrients found tweets mentioning general nutrients, sodium restriction, energy needs, dietary supplements, alcohol, and proteins for heart failure [15]. This study only collected tweets that contained the “#heartfailure” hashtag, which resulted in a limited number of tweets and did not provide a comprehensive understanding of tweets related to nutrients and foods for blood pressure control [15]. Furthermore, these studies did not investigate the user characteristics that share information about nutrients and foods for managing hypertension.

Content related to hypertension, nutrients, and food is highly prevalent on Twitter, indicating that this topic interests the public. To our knowledge, no study has analyzed the content of tweets related to nutrients and foods for blood pressure control on Twitter. Therefore, this study aimed to investigate the nature of the information people are exposed to on Twitter regarding nutrients and foods for blood pressure control. Furthermore, we discuss the potential impact of this information on Twitter users’ dietary behaviors. The research questions (RQs) are as follows:

- RQ1: What types of nutrients and foods are recommended on Twitter for blood pressure control?
- RQ2: Do the content of tweets related to blood pressure control, nutrients, and food vary according to user characteristics?
- RQ3: What types of foods, nutrients, and themes are commonly featured in the highly retweeted tweets related to blood pressure control, nutrients, and foods?

## Methods

### Data Extraction

**Figure 1:**
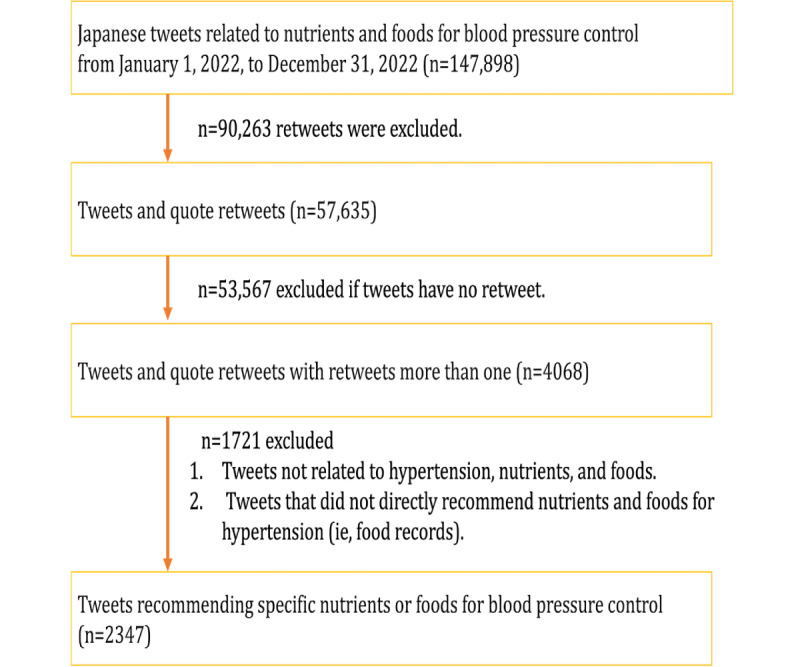
Flow diagram for inclusion and exclusion of tweets posted from January 1, 2022, to December 31, 2022.

We extracted 147,898 Japanese tweets posted from January 1, 2022, to December 31, 2022, matching keywords related to hypertension, nutrients, and food consumption. The keywords used in the search are listed in Multimedia Appendix 1, as we used complex Japanese terms. The keywords were selected based on previous hypertension studies on Facebook [14] and a content analysis of tweets related to heart failure and nutrients on Twitter [15]. The data were obtained from Social Insight [16], a subscription Twitter aggregation service that allows the purchase of tweets and their associated data, such as the number of retweets likes, and followers. We obtained tweet texts, user descriptions, usernames, number of retweets, likes, followers, and posted times. As Figure 1 shows, retweets were excluded from 147,898 tweets, leaving 57,635 eligible for analysis. The Pearson correlation coefficient (*r*) between the daily number of tweets and the daily number of retweets was calculated to be 0.28 (95% CI 0.19-0.37), indicating a low correlation between the number of tweets and their diffusion to the public, as measured by retweet counts. Accordingly, we assumed that sampling based on tweet counts for a certain period might not capture tweets with a high diffusion index, as measured by retweet counts. Therefore, to analyze the tweets more likely to have been exposed to a larger audience, we conducted purposive sampling by focusing on tweets with high retweet counts in our study sample. After calculating the retweet counts of the 57,635 tweets, we found that the lower quartile value for retweet was 0 up to the 75th percentile. Limiting the analysis to tweets with at least 1 retweet (ie, retweet counts above the 75th percentile), we extracted 4068 tweets. Next, we manually screened 4068 tweets based on the following inclusion and exclusion criteria: the inclusion criteria were tweets recommending specific nutrients or foods for blood pressure control. The exclusion criteria were tweets unrelated to hypertension, nutrients, or foods, or tweets that did not directly recommend nutrients or foods for blood pressure control (ie, personal food records). We included 2347 tweets in the final analysis.

### Coding Procedures

#### Procedure and Data Analysis

The first author (MT) screened 4068 tweets with the aforementioned exclusion criteria and created a coding manual. Subsequently, 2347 tweets were deemed eligible for final coding. Then, MT conducted a training session using a manual with the third author (RY). MT and RY coded 469 tweets (20% of the final sample of 2347 tweets) that were randomly sampled to assess interrater reliability for all categories. If there were discrepancies between the coders, a second author (TO) joined the discussion until a consensus was reached. Excel (version 16.70; Microsoft Corp) was used for coding.

#### User Characteristics

Based on the usernames and self-descriptions, we coded users as “health, losing weight, and beauty-related users,” “food manufacturer,” “IT company and mass media,” “government and academic institution,” “medical institution,” and “pharmaceutical manufacturer” based on our previous study [7]. Subsequently, we inductively added user characteristics [17]. If a user matched 2 or more attributes, they were categorized into all applicable categories (eg, 1 user could be a doctor and food and nutrition-related users). Users not matching all the listed attributes were classified as “general public.”

#### Themes of Tweets

Tweets containing recommendations for foods that can help control blood pressure were categorized into food groups using the Food Composition Database provided by the Japanese Ministry of Education, Culture, Sports, Science, and Technology [18]. In a previous study, there have been reports suggesting the existence of misleading information claiming that salt reduction has no effect on blood pressure management or advocating alternative treatments not recommended by current guidelines [19]. Building upon these findings, this study also categorized misleading information that denies the relationship of salt reduction for hypertension or advocating alternative measures not recommended by current guidelines. The nutrients were added inductively. Each food or nutrient mentioned in the tweets was added to its respective category. Because this study aimed to analyze the content of the tweets, we did not code the web pages linked to the URLs in the tweets. Images (eg, photographs) containing information about foods or nutrients recommended for blood pressure control were considered for analysis. As an indicator of credibility, we coded the presence or absence of citations to support the information provided in the tweet. To code the citation sources, we adopted a deductive approach for scientific papers, books, dietary reference intakes, the Ministry of Health, Labour and Welfare, the Japanese Society of Hypertension, the Japanese Circulation Society, and the Dietary Approaches to Stop Hypertension, as described in our previous study [7]. We also used an inductive approach to include other sources, such as specific health food products approved by the Consumer Affairs Agency in Japan and news articles. We classified tweets as advertisements if they promoted a specific brand, product, or store for advertising purposes, such as recommending a particular brand or product for blood pressure control.

### Statistical Analysis

The number and percentage of tweets, retweets, and tweets in each category were calculated. To evaluate the characteristics of the highly diffused tweets, they were classified into 4 quartiles based on the number of retweets. The number and percentage of tweets in each category are shown. The interrater reliability coefficient was calculated using the Gwet AC1 statistic, which is more consistent with a lower prevalence than Cohen κ statistics [20-22]. The analysis was conducted using R for macOS (version 4.2.0; The R Foundation).

### Ethical Considerations

This study was approved by the ethics committee of the Graduate School of Medicine at the University of Tokyo (2022288NI). Inform consent could not be obtained due to the nature of the data. This report does not contain any identifying information or direct quotes in accordance with published guidelines and recommendations to support anonymity [23].

## Results

### Sample Description

The final sample consisted of 2347 Japanese tweets that met the inclusion criteria, posted from January 1, 2022, to December 31, 2022, with more than 1 retweet. The number of unique users was 1497 after removing duplicate accounts.

### Nutrients and Foods Recommended for Blood Pressure Control

Table 1 shows the types of food groups and nutrients recommended for blood pressure control in the tweets. The interrater reliability was from 0.80 (95% CI 0.75-0.86) to 1.00 (95% CI 1.00-1.00) for these categories. In the analysis of 2347 tweets, 80% (n=1877) of tweets mentioned foods, which were categorized into 17 different food groups. Among food groups, seasonings and spices accounted for the largest proportion of tweets at 72.2% (n=1356), of which, tweets mentioning salt accounted for a large proportion at 69.3% (n=1301), representing 55.4% (n=1301) of the total tweets analyzed. A food group of vegetables accounted for 13.6% (n=256), followed by fruits at 4.4% (n=83) and seafood at 2.9% (n=55). Of 2347 tweets analyzed, 1566 (66.7%) mentioned nutrients, specifically 15 different types of nutrients recommended for blood pressure control. Sodium was the most frequently discussed nutrient at 83.1% (n=1301), followed by potassium at 8.4% (n=132) and minerals at 4.7% (n=73). Among the nutrients mentioned, 1.3% (n=21) referenced dietary supplements, with 71.4% (n=15) of these mentions specifically relating to γ-aminobutyric acid (Multimedia Appendix 2).

**Table 1:** The types of foods and nutrients recommended for blood pressure control in tweets posted from January 1, 2022, to December 31, 2022 (N=2347)^a^.

		Values, n (%)
**Food groups (n=1877)**		
	Seasonings and spices	1356 (72.2)
	Vegetables	256 (13.6)
	Fruits	83 (4.4)
	Fish and shellfish	55 (2.9)
	Beverages	43 (2.3)
	Cereals	40 (2.1)
	Potatoes	29 (1.5)
	Pulses	28 (1.5)
	Dairy products	26 (1.4)
	Nuts and seeds	24 (1.3)
	Mushrooms	13 (0.7)
	Algae	12 (0.6)
	Meat	9 (0.5)
	Confectioneries	8 (0.4)
	Fats and oils	3 (0.2)
	Sugars and sweeteners	3 (0.2)
	Ready-to-eat foods	119 (6.3)
**Nutrients (n=1566)**		
	Sodium^b^	1301 (83.1)
	Potassium	132 (8.4)
	Minerals	73 (4.7)
	Protein	61 (3.9)
	Dietary fiber	51 (3.3)
	Magnesium	49 (3.1)
	GABA^c^	28 (1.8)
	Citric acid	25 (1.6)
	Sodium bicarbonate	22 (1.4)
	Calcium	22 (1.4)
	EPA^d^	16 (1)
	Omega-3 fatty acids	14 (0.9)
	DHA^e^	12 (0.8)
	Iron	12 (0.8)
	Polyphenols	10 (0.6)
	Others (eg, zinc and linoleic acid)	11 (0.7)

^a^A single tweet can be classified into multiple foods and nutrients, and the total number of tweets may exceed the total number of unique tweets. Food group of eggs was excluded, as they were not recommended for blood pressure control. Carbohydrates were mentioned in 66 tweets but were excluded because they were not recommended for blood pressure control.

^b^Salt was included with sodium as it is commonly tweeted about inseparably and interchangeably.

^c^GABA: γ-aminobutyric acid.

^d^EPA: eicosatetraenoic acid.

^e^DHA: docosahexaenoic acid.

### Themes of Tweets and User Characteristics

Table 2 presents the proportion of tweets related to each theme based on user characteristics, definitions, and the total number of tweets associated with each user characteristic. The interrater reliability was from 0.84 (95% CI 0.79-0.88) to 1.00 (95% CI 1.00-1.00) for these categories. The users were classified into 12 categories. Except for the general public at 70.6% (n=1658), advertisement users, such as e-commerce users, accounted for the largest proportion of tweets at 10.3% (n=242), followed by “health, losing weight, and beauty-related users” at 10.1% (n=238), “food and nutrition-related users” at 6.9% (n=163), 69%-93.6% of users recommended any food for blood pressure control.

**Table 2:** The user characteristics, the number of tweets and themes, posted from January 1, 2022, to December 31, 2022.

User characteristics	Definition	Tweets (n=2347), n (%)^a,b^	Recommendation for blood pressure control			Themes of tweets			
			Foods (n=1877), n (%)^c^	Nutrients (n=1566), n (%)^c^	Prosalt reduction (n=770), n (%)^c^		Antisalt reduction (n=531), n (%)^c^	Advertisement (n=242), n (%)^c^	Reference (n=122), n (%)^c^
Nutritionist or registered dietitian	Nutritionist or registered dietitian	17 (0.7)	15 (88.2)	12 (70.6)	5 (29.4)		4 (23.5)	1 (5.9)	2 (11.8)
Doctors	Doctors	28 (1.2)	23 (82.1)	24 (85.7)	21 (75)		2 (7.1)	2 (7.1)	8 (28.6)
Other medical professionals	Veterinarian, dentist, dental hygienist, nurse, midwife, public health nurse, pharmacist, clinical psychologist, caregiver welfare worker, acupuncturist, and Chinese herbalist	58 (2.5)	40 (69)	33 (56.9)	11 (19)		8 (13.8)	2 (3.4)	1 (1.7)
Health, losing weight, and beauty-related users	Users who clearly indicate that they tweet about health, hypertension, losing weight, and beauty	238 (10.1)	196 (82.4)	150 (63.0)	26 (10.9)		74 (31.1)	26 (10.9)	8 (3.4)
Food and nutrition-related users	Users who clearly indicate that they tweet about cooking, meals, and nutrition or those who described themselves as food coordinator and supplement adviser	163 (6.9)	135 (82.8)	77 (47.2)	30 (18.4)		12 (7.4)	25 (15.3)	16 (9.8)
Food manufacturer	Food manufacturers such as soba food companies	47 (2)	44 (93.6)	28 (59.6)	22 (46.8)		1 (2.1)	19 (40.4)	5 (10.6)
IT company and mass media	Official news media and digital news media	42 (1.8)	31 (73.8)	24 (57.1)	20 (47.6)		2 (4.8)	1 (2.4)	7 (16.7)
Government, organizations, and university institutions	Government, organizations, and university institutions	31 (1.3)	28 (90.3)	26 (83.9)	21 (67.7)		3 (9.7)	2 (6.5)	0 (0)
Medical institutions	Hospitals and clinics	15 (0.6)	12 (80)	12 (80)	10 (66.7)		1 (6.7)	0 (0)	6 (40)
Pharmaceutical manufacturer	Pharmaceutical manufacturers	40 (1.7)	35 (87.5)	25 (62.5)	9 (22.5)		4 (10)	2 (5)	2 (5)
Advertisement	Digital shopping companies and other accounts that promote their products for advertisement purposes	242 (10.3)	219 (90.5)	163 (67.4)	118 (48.8)		8 (3.3)	91 (37.6)	20 (8.3)
General public	Users could not be classified into any of the above	1658 (70.6)	1299 (78.3)	1115 (67.2)	531 (32)		429 (25.9)	276 (16.6)	67 (4)

^a^An account can be classified into multiple user characteristics, and the total number of tweets in each column may exceed the total number of unique tweets.

^b^The percentage in the tweet count column is based on 2347 tweets and shows the distribution of each user attribute in the total number of tweets.

^c^Percentage is based on the total tweet count for each user and shows the distribution of themes for each user.

Of the total tweets analyzed, 1301 (55.4%) referred to salt. These tweets included 2 opposing views on reducing salt intake in hypertension control. The “prosalt reduction theme” recommended reducing salt intake for hypertension control, while the “antisalt reduction theme” claimed that reducing salt intake was not related to higher blood pressure or that consuming salt was beneficial for lowering blood pressure. Among tweets referred salt, 59.2% (n=770) was the “prosalt reduction theme” and 40.8% (n=531) was the “antisalt reduction theme.” Regarding the relationship between user characteristics and the theme of salt reduction, 75% (n=21) of tweets from “doctors” and 66.7% (n=10) from “medical institution” mentioned salt reduction is effective for hypertension control. In contrast, 31.1% (n=74) of tweets from “health, losing weight, and beauty-related users,” 25.9% (n=429) of tweets from “general public,” and 23.5% (n=4) tweets from “dietitian or registered dietitian” denied salt reduction for hypertension control.

Regarding advertising tweets, 40.4% (n=19) of “food manufacturer,” 37.6% (n=91) of “advertisements users,” 16.6% (n=276) of “general public,” and 15.3% (n=25) of “food and nutrition-related users” had posted tweets for advertising purposes. Regarding the inclusion of citations, 40% (n=6) of medical institutions and 28.6% (n=8) of doctors included references in their tweets.

### Number of Retweets and Themes

The median number of retweets was 2.0 (IQR 1.0-7.0), with a maximum of 7292. Among the 2347 tweets, the 0th percentile and 25th percentile of retweets were both equal to 1. Consequently, tweets with only 1 retweet were excluded to categorize tweet counts based on the quartiles of retweet numbers, as shown in Table 3. Among tweets with more than 2 retweets (n=1372), there were 530 tweets in the first quartile (1Q), 184 tweets in the second quartile (2IQR), 321 tweets in the third quartile (3IQR), and 337 tweets in the fourth quartile (4IQR). Among tweets in the lowest 1Q of retweet numbers, the percentage of “prosalt reduction theme” for hypertension was 31.7% (n=168); however, this percentage decreased to 24.9% (n=84) among tweets in the highest 4IQR of retweet numbers. Conversely, among tweets in the lowest 1Q of retweet numbers, the percentage of “antisalt reduction theme” for hypertension was 22.6% (n=120); this percentage increased to 31.5% (n=106) among tweets in the highest 4IQR of retweet numbers.

**Table 3:** The distribution of each category in tweets classified by retweet quartiles posted from January 1, 2022, to December 31, 2022 (n=1372)^a^.

Retweet	Median (IQR)	Range	Unique tweets, n	Recommendation for blood pressure control			Themes of tweets			
				Foods, n (%)	Nutrients, n (%)	Prosalt reduction, n (%)		Antisalt reduction, n (%)	Advertisement, n (%)	Reference, n (%)
1Q	2.0 (2.0-3.0)	2.0-3.0	530	418 (78.9)	342 (64.5)	168 (31.7)		120 (22.6)	55 (10.4)	31 (5.8)
2IQR	4.0 (4.0-5.0)	4.0-5.0	184	147 (79.9)	120 (65.2)	57 (31)		47 (25.5)	12 (6.5)	10 (5.4)
3IQR	9.0 (7.0-12.0)	6.0-17.0	321	237 (73.8)	186 (57.9)	77 (24)		77 (24)	30 (9.3)	27 (8.4)
4IQR	57.0 (28.0-166.0)	18.0-7292.0	337	288 (85.5)	238 (70.6)	84 (24.9)		106 (31.5)	22 (6.5)	16 (4.7)

^a^Because of the possibility of each tweet being classified into more than 1 category, the total may not add up to the total number of tweets analyzed. The percentage denominator represents the unique number of tweets in each quartile category and shows the distribution of themes in each quartile.

Multimedia Appendix 3 shows the tweet content of the top 15 tweets with the most retweets among the total sample of 2347 tweets. The first 6 tweets were disseminated by users categorized as food company and advertisement user, promoting their products for blood pressure control. The 8th, 12th, and 15th tweets contained antisalt reduction content, endorsing natural salt or asserting that increased salt intake is beneficial for lowering blood pressure. The 9th and 10th tweets attempted to debunk such antisalt reduction content. Other tweets in Multimedia Appendix 3 promoted various products or specific vegetables.

## Discussion

### Principal Results

This study obtained 147,898 tweets that matched keywords related to hypertension, nutrients, and foods, including retweets, posted on Twitter in 2022. Of these, 2347 tweets were analyzed after purposive sampling, and the recommended nutrients and foods for blood pressure control, themes, and user characteristics were identified. The large number of tweets in this study indicates a high level of interest in nutrients and foods for blood pressure control. However, in this study, 40.8% (n=531) of tweets asserted that salt reduction has no effect on blood pressure management or endorsed alternative treatments not recommended by current guidelines. Moreover, more than 30% (106/337) of the most disseminated tweets were antisalt reduction, suggesting the spread of misinformation of salt for blood pressure control.

### Nutrients and Foods Recommended for Blood Pressure Control

In total, 80% (n=1877) of all tweets mentioned foods for hypertension control. The most mentioned food groups were seasonings and spices, followed by vegetables, fruit, and seafood. Salt was the most frequently mentioned item in both nutrients and food groups, accounting for more than half of all tweets in the analysis. These results are consistent with scientifically recommended food groups for preventing and controlling hypertension [5,6,24]. Potassium is the second frequently mentioned nutrient and is consistent with the recommended nutrients for blood pressure control [5]. There is less mention of recommended nutrients for blood pressure control, such as reducing saturated and total fats [5,6,25] than minerals, which were not specified in the tweets.

### Themes of Tweets and User Characteristics

The majority of tweets (n=1658, 70.6%) were from the “general public,” which could not be classified, showing the anonymity of Twitter. This was followed by “advertisement users,” “health, losing weight, and beauty-related users,” and “food and nutrition-related users.” This study found that users who identified themselves as health gurus or health or diet experts, as previously noted in other studies [26], were among the most frequent sources of highly disseminated tweets regarding food and nutrients for blood pressure control in addition to general public. In other words, people are exposed to information about nutrients and foods for blood pressure control on Twitter primarily from users who identify themselves as health gurus or health or diet experts rather than from medical professionals, registered nutritionists, or medical institutions. Furthermore, more than 30% (n=74) of “health, losing weight, and beauty-related users” promoted increased salt intake for lowering blood pressure. A reduction in dietary salt intake reduces blood pressure [27,28], which is dose-dependent and has a linear relationship rather than a J-shaped one [29]. However, this study found that self-proclaimed experts on Twitter [26] spread misleading information on nutrients and foods for blood pressure control. In recent years, several studies suggesting that reducing salt intake is ineffective in treating hypertension have been published in a single journal. Several biases have been pointed out, such as funding from the food industry, raising concerns about the reliability, and ethics of science [30]. This study further suggests that misinformation about salt reduction for hypertension is disseminated on social media platforms such as Twitter. Additionally, although registered dietitians and nutritionists are expected to combat misinformation about nutrition [26], in this study, the number of tweets from registered dietitians or nutritionists was only 0.7% (n=17). Furthermore, 23.5% (n=4) of them disseminated misleading information that denied an association between excessive salt intake and hypertension, contradicting the guidelines for hypertension and diet [5,6]. Conversely, 75% (n=21) of doctors recommended reducing salt intake, but the number of tweets from doctors was only up to 1.2% (n=28); it is difficult to evaluate whether they could combat misinformation about the controversial discussion about salt reduction. Cappuccio et al [30] have provided a matrix of facts that refute myths skeptical of reducing salt intake, such as “our body needs salt and restriction is a mistake” or “natural salt is healthier than refined salt,” which aligns with the content in this study. Therefore, nutritionists, registered dietitians, and doctors should use this matrix to combat misinformation and proactively provide accurate information on salt intake and hypertension control.

Although “medical institutions” and “doctors” cited references frequently, only 5.2% (n=122) of tweets overall included citations. This may be because of the limit of 140 Japanese characters per tweet. Previous research showed that at least 40% of nutrition-related websites included 1 or more citations [7], suggesting that the low citations on Twitter make it difficult to select reliable information for lay individuals.

### Number of Retweets and Themes

This study found that antisalt reduction tweets accounted for 31.5% (n=106) of the most disseminated tweets related to nutrients and food for blood pressure control, whereas only 24.9% (n=84) were prosalt reduction tweets. This result is consistent with previous studies showing that fake news is more likely to spread than true news [31], and misleading videos on hypertension have been found to have high user engagement [19]. However, studies examining the credibility of tweets have reported that the more retweets a tweet has, the lower the reader’s trust in it [32-34]. Therefore, there is a possibility that the credibility of antisalt reduction with a high number of retweet is low, although these studies did not evaluate the credibility of tweets related to nutrition and hypertension [35]; it is not clear how the number of retweet for nutrition-related tweets is related to their credibility by Twitter users, which requires further investigation. Furthermore, while retweets are commonly used as a measure of diffusion [31,36], they also serve as a means to “argue” against the content being retweeted [37,38]. Hence, it is crucial to understand that retweets against antisalt reduction might also involve sharing content to counteract it. The dissemination of misinformation through tweets with high retweet numbers and greater exposure is a cause for concern. Evaluating the details of misinformation regarding salt reduction on Twitter and its potential impact on public dietary habits is necessary.

### Limitations

This study had some limitations. First, user characteristics were obtained from the self-description profile and usernames and may not represent the actual status. Next, blood pressure increases with age [39,40], a trend that may not align with the predominantly young age group using Twitter [10]. However, hypertension should be addressed through a life-course approach that includes younger age groups [41]. Therefore, analyzing and evaluating information related to nutrients and foods as modifiable factors of hypertension on Twitter, which is a widely used information channel in Japan for young and older people, are important. Third, this study used the number of retweets to indicate dissemination and influence without considering the number of followers or favorites. Fourth, because this study is a content analysis that enables the evaluation of communication symbols, we could not evaluate the influence of exposure to tweets on public perception and actual eating behavior. Finally, the tweets analyzed in this study were limited to Japanese; information from other languages was not investigated. Japanese cultural foods, such as miso, which are unique to Japan, were recommended for preventing hypertension in this study, indicating cultural characteristics. In future research, it will be necessary to conduct studies that include multiple languages and examine the kinds of information related to nutrients and foods recommended for hypertension control that are being disseminated in the context of different cultures.

### Conclusions

This study identified the recommended nutrients and foods for blood pressure control and user characteristics on Twitter in 2022. The food groups and nutrients recommended in the tweets were consistent with the guidelines, but misinformation related to salt reduction accounted for more than 40.8% (n=531) of the tweets referred salt for blood pressure control. Most of the misinformation was tweeted by users including general public and self-proclaimed health experts. The number of tweets from nutritionists, registered dietitians, and doctors who were expected to correct misinformation and promote salt reduction was relatively low, and their messages were not always positive toward salt reduction. There is a need for communication strategies to combat misinformation, promote correct information on salt reduction, and train health care professionals to effectively communicate evidence-based information on this topic.

## Appendix

Multimedia Appendix 1 The keywords selected for searching tweets related to nutrients and foods for blood pressure control.

Multimedia Appendix 2 The proportions of tweets about nutrients that include supplements posted from January 1, 2022, to December 31, 2022.

Multimedia Appendix 3 Top 15 tweets with the most retweets posted from January 1, 2022, to December 31, 2022.

## Abbreviations

1Q: first quartile
2IQR: second quartile
3IQR: third quartile
4IQR: fourth quartile
RQ: research question

## Data Availability

The data sets generated and analyzed during this study are not publicly available as they were purchased from the Social Insights. However, they are available from the corresponding author on reasonable request with permission from the Social Insights.
