# Supplementary material for: Nutrients and Foods Recommended for Blood Pressure Control on Twitter in Japan: Content Analysis
Source: J Med Internet Res. 2024 Jun 20;26:e49077. doi: 10.2196/49077 (PMC11224700; doi:10.2196/49077)
Supplement: Multimedia Appendix 1 [file jmir_v26i1e49077_app1.docx]

| Final Keywords | 「(血圧 OR 降圧) (食 OR 栄養 OR 摂 OR 飲 OR 料理 OR 飯 OR ごはん OR サプリ OR メニュー OR 塩 OR アルコール OR 酒)」 | | |
| --- | --- | --- | --- |
| Keywords | Translation | Keywords | Translation |
| 血圧^a^ | blood pressure | 降圧 | lowering blood pressure |
| 食 | food | 栄養 | nutrient |
| 摂^b^ | intake | 飲 | drink |
| 料理 | cooking | 飯 | meal |
| ごはん | diet | サプリ^c^ | supplement |
| メニュー | menu | 塩 | salt |
| アルコール | alcohol | 酒 | *sake^d^* |

a Due to the nature of tweet data, Japanese terms of “血圧” can extract all keywords of high blood pressure, hypertension and lowering blood pressure.

b Due to the nature of tweet data, Japanese terms of “摂” can extract the word of intake including a Japanese word such as “摂取” and “摂る”.

c Due to the nature of tweet data, Japanese terms of “サプリ” can extract the word of intake including both Japanese word of “サプリメント” and “サプリ”.

d In Japanese, there are another term of “sake” for expressing alcohol.
