# Supplementary material for: Nutrients and Foods Recommended for Blood Pressure Control on Twitter in Japan: Content Analysis
Source: J Med Internet Res. 2024 Jun 20;26:e49077. doi: 10.2196/49077 (PMC11224700; doi:10.2196/49077)
Supplement: Multimedia Appendix 2 [file jmir_v26i1e49077_app2.docx]

| Nutrients | n | Supplement | % | |
| --- | --- | --- | --- | --- |
| Sodium^a^ | 1301 | 0 | 0.0 | |
| Potassium | 132 | 2 | 9.5% | |
| Minerals | 73 | 0 | 0.0% | |
| Protein | 61 | 0 | 0.0% | |
| Dietary fiber | 51 | 0 | 0.0% | |
| Magnesium | 49 | 1 | 4.8% | |
| GABA^b^ | 28 | 15 | 71.4% | |
| Citric acid | 25 | 0 | 0.0% | |
| Sodium bicarbonate | 22 | 0 | 0.0% | |
| Calcium | 22 | 0 | 0.0% | |
| EPA^c^ | 16 | 1 | 4.8% | |
| Omega-3 fatty acids | 14 | 1 | 4.8% | |
| DHA^d^ | 12 | 1 | 4.8% | |
| Iron | 12 | 0 | 0.0% | |
| Polyphenols | 10 | 0 | 0.0% | |
| Others (i.g. Zinc, Linoleic acid) | 11 | 0 | 0.0% | |
| Total | 1566 | 21 |  | |
| ^a^ Salt was included with sodium as it is commonly tweeted about inseparably and interchangeably. | | | |  |
| ^b^ GABA = gamma-aminobutyric acid | | | |  |
| ^c^ EPA = eicosatetraenoic acid | | | |  |
| ^d^ DHA = docosahexaenoic acid | | | |  |
