# Supplementary material for: Nutrients and Foods Recommended for Blood Pressure Control on Twitter in Japan: Content Analysis
Source: J Med Internet Res. 2024 Jun 20;26:e49077. doi: 10.2196/49077 (PMC11224700; doi:10.2196/49077)
Supplement: Multimedia Appendix 3 [file jmir_v26i1e49077_app3.docx]

| No. | User Characteristics | Tweet Content | The number of retweets | The number of likes |
| --- | --- | --- | --- | --- |
| 1 | Food manufacturer & Advertisement | Follow us and RT to win a set of low-sodium snacks! May 17th is #WorldHypertensionDay and 17th of every month is #DecreaseSaltDay. Would you like to start a healthy life with less salt? How to participate ①Follow ②RT this post by May 18 11:59 ③Winners will be sent by DM | 7292 | 1513 |
| 2 |  |  | 6791 | 1175 |
| 3 |  |  | 6314 | 1034 |
| 4 |  |  | 5909 | 988 |
| 5 |  |  | 5549 | 886 |
| 6 | Food company & Advertisement | Yogurt Health Checkup Support Campaign. Tell us what you are interested in with the hashtag. 100 people will win a month supply of healthy yogurt. ①Follow ②One of #ElevatedBloodPressure, #PostprandialBloodSugar, or #PostprandialNeutralFat, and tweet a quote from this post. | 4955 | 5455 |
| 7 | Food and nutrition-related users | I'm sure you all know that "Vinegared Daikon" is really recommended if you want to lose internal fat. Vinegar helps to burn fat and normalize blood pressure, and the seasonal green neck daikon helps to regulate the stomach and intestines. 1) Cut daikon into bite-size pieces, 2) Rub with salt, 3) Add kombu or niboshi to the vinegar and broth and let it marinate for a while and you're done. | 4428 | 30471 |
| 8 | General Public | Fake salt is making you sick. Sodium chloride, which has been produced by the ion-exchange membrane salt process since 1971, is just salty and not real salt. This is the salt we normally use at the dinner table. Consumption of fake salt causes high blood pressure and a lowered immune system. Switch to rock salt or sea salt. | 3029 | 11701 |
| 9 | Doctors | The person known as the 'Salt Man' may seem like a joke, but the reason medical professionals view him with concern is that while typical pseudo medical advice often recommends 'ineffective measures,' he advocates for 'directly harmful actions' by recommending large amounts of salt intake to individuals with conditions like high blood pressure and renal dysfunction. | 2332 | 5931 |
| 10 | General Public | I'm shocked. "Sugar turns children into demons," "Natural salt sings in the body," "Reduced salt is a form of punishment," "Natural salt turns into magnesium through atomic conversion," "Reduced salt raises blood pressure," "Natural salt lowers blood pressure," "Thank you makes my cells happy," I've never heard any of these. I never learned them in medical school. I've never read a scientific paper. I want to know the evidence. | 2318 | 7665 |
| 11 | Food and nutrition-related users | A request from a potherb mustard farmer. When your intestines are weak or your blood pressure is high and you feel lightheaded, I highly recommend "Boiled-Tofu with wakame seaweed and potherb mustard". The stickiness of the wakame seaweed will help to regulate the intestines, and the rich potassium in the potherb mustard will help with diuresis and salt elimination. Let's replenish our body and mind with nourishment and kindness by eating potherb mustard, which is 30% cheaper than usual. | 2109 | 7569 |
| 12 | Health, losing weight and beauty-related users | Many people think that eating salt raises blood pressure, but it's actually the opposite. Blood pressure rises because you don't eat salt. When blood pressure drops due to lack of salt, capillaries are unable to pump blood. Since it would be fatal, the heart muscle is enlarged to increase the pressure in the blood vessels to supply nutrients. This is the mechanism of hypertension and myocardial hypertrophy. This is not the time to be cutting back on salt. | 1674 | 6004 |
| 13 | General Public | This is a tough one. To lower blood pressure, "reduce alcohol," "reduce excess weight," "reduce salt," "get potassium," "get vegetables," "get fruits," "but even more potassium, vegetables, and fruits won't cancel out the other effects." | 1511 | 6043 |
| 14 | General Public | I am neither a dietician nor a doctor, but I may know how to cure an allergic disease overnight. If you eat at least 100 grams of burdock for dinner, you will be cured the next morning. This is because the fructooligosaccharides in burdock increase Treg cells. Diabetes and high blood pressure are also cured. | 1468 | 5933 |
| 15 | Doctors & Medical institutions | High blood pressure is not bad, and Japanese people have always had high blood pressure. Salt is being demonized, but it is not the salt that is bad, it is the refined salt that is the problem. Real salt contains micro minerals and is necessary for Japanese people. The lower the blood pressure, the more likely you are to get cancer, dementia, infectious diseases, etc. | 1378 | 6110 |
| Tweet No.9 mentioned the 'Salt Man,' referring to user No.12, who was the second most active tweeter in this sample (n=43/2347) in 2022, with a primary focus on anti-salt reduction theme. | | | | |
